# Experiences of intimate partner violence and valued living among women veterans: The role of self-efficacy

**DOI:** 10.1002/jts.23059

**Published:** 2024-06-11

**Authors:** Emily Taverna, Nora Kline, Shaina A. Kumar, Katherine M. Iverson

**Affiliations:** 1 Women’s Health Sciences Division of the National Center for PTSD, Boston, Massachusetts, USA; 2 VA Boston Healthcare System, Boston, Massachusetts, USA; 3 Department of Psychiatry, Boston University Chobanian & Avedisian School of Medicine, Boston, Massachusetts, USA; 4 Behavioral Science Division of the National Center for PTSD, Boston, Massachusetts, USA

## Abstract

Predominantly cross-sectional research suggests that self-efficacy may play an important role in women’s psychological health after experiencing intimate partner violence (IPV). However, few studies have examined these associations over time or with respect to broader aspects of psychological well-being. Valued living, which reflects behavioral engagement within personally important life domains, represents a key aspect of well-being that may be negatively impacted by experiences of IPV. Participants were 190 women veterans who completed three web-based surveys. We examined whether IPV experiences at Time 1 were associated with valued living at Time 3 (i.e., 4 years after Time 1) through self-efficacy at Time 2 (i.e., 3 years after Time 1). We separately examined overall, psychological, physical, and sexual IPV and investigated lifetime and recent (i.e., past 6 months) IPV experiences for each subtype. Separate path analysis models indicated that lifetime overall, *β* = −.10, 95% CI [−.19, −.02]; psychological, *β* = −.08, 95% CI [−.17, −.001]; physical, *β* = −.10, 95% CI [−.18, −.01]; and sexual, *β* = −.11, 95% CI [−.22, −.01], IPV experiences were indirectly associated with less valued living through less self-efficacy, whereas the indirect effect only emerged for recent physical IPV, *β* = −.26, 95% CI [−.50, −.02], but not for recent overall, psychological, or sexual IPV. These findings suggest that experiencing IPV is associated with less self-efficacy and valued living, which highlights the importance of providing early psychosocial interventions to enhance well-being among individuals managing the effects of experiencing IPV.

Intimate partner violence (IPV) includes verbal remarks, threats, or behaviors used by a current or past intimate partner to cause psychological, physical, and/or sexual harm (World Health Organization [WHO], 2021). IPV disproportionately impacts women; one third of women worldwide experience IPV during their lifetime. In the United States, at least one in three women experience physical violence, sexual coercion, or stalking by an intimate partner during their lifetime (Smith et al., 2018). Additionally, psychological IPV is common, with nearly half (49%) of women in the U.S. experiencing psychological IPV in their lifetime (Smith et al., 2018). IPV experiences have widespread detrimental effects on women’s well-being, including physical and mental health concerns, occupational and financial difficulties, and reduced social support (e.g., Bonomi et al., 2006; Peterson et al., 2018). To inform intervention targets, research is needed to identify modifiable factors that contribute to reduced well-being among women who experience IPV.

*Valued living* is one important transdiagnostic component of broader well-being that spans multiple life domains (e.g., health, work, relationships) and may be negatively affected by experiencing IPV. *Values* refer to idiosyncratic and intrinsically meaningful frameworks, and valued living refers to the extent to which individuals behaviorally engage with their personal values in everyday life (Wilson et al., 2010). Prior research suggests that valued living is associated with other components of well-being. Specifically, longitudinal research suggests that some aspects of well-being (e.g., a sense of meaning, positive emotion, life satisfaction) are positively associated with valued living and that valued living may help maintain positive well-being over time (Finkelstein-Fox et al., 2020; Kashdan et al., 2006; Williams et al., 2015). As such, valued living represents an important behavioral component of positive psychological health.

Despite the theoretical relevance of valued living for women with IPV experiences (Iverson et al., 2021), this construct has been minimally examined among this population. IPV is often chronic, and managing the direct effects and secondary consequences of IPV may lead to significant stress and trauma even after abusive relationships have ended (Cerulli et al., 2012; Hing et al., 2021). This is cause for concern given that prior cross-sectional (Ceary et al., 2019; Donahue et al., 2017) and longitudinal (Finkelstein-Fox et al., 2020) research suggests that elevated stress is associated with reduced valued living and that the ability to maintain greater valued living may buffer the negative effects of stressful life events on overall resilience and functioning. Accordingly, understanding modifiable factors that promote or inhibit valued living may inform important targets for IPV intervention.

Research on the mental health consequences of IPV has often emphasized psychological disorders, such as posttraumatic stress disorder (PTSD) and depression (see Spencer et al., 2019, for a review). Examining broader aspects of psychological health common to many psychological disorders may shed light on important transdiagnostic mechanisms and outcomes. For example, a cross-sectional study among young adult interpersonal violence survivors, including but not limited to IPV, found that trauma-related shame was indirectly linked to increased obstacles to pursuing values and reduced progress toward values above and beyond the contribution of PTSD symptoms (Cabrera et al., 2021). These findings underscore the importance of moving beyond traditional diagnostic indices of mental health symptoms to examine broader transdiagnostic aspects of well-being. Additional longitudinal research is needed to elucidate psychological processes associated with valued living, particularly among women who have experienced IPV.

Self-efficacy is understudied as a transdiagnostic process that may influence valued living. Social cognitive theory refers to self-efficacy as the belief that one can cope with difficult life experiences, and self-efficacy is thought to influence individuals’ behavioral choices, motivation, and effort across a range of life domains (Bandura, 1977; Schunk & DiBenedetto, 2021). Cross-sectional and longitudinal research suggests that higher levels of self-efficacy may buffer the effects of stress and trauma on negative mental health symptoms (e.g., PTSD, depression; see Gallagher et al., 2020, and Luszczynska et al., 2009, for reviews). Additionally, self-efficacy has demonstrated positive cross-sectional and longitudinal associations with broader aspects of positive psychological health, such as life satisfaction and optimism (Azizli et al., 2015; Schönfeld et al., 2016). Together, this suggests that self-efficacy may play an important role in the maintenance of well-being, particularly in the context of stress. A small body of literature has examined the impact of self-efficacy on psychological symptoms among IPV survivors given that qualitative (Burke et al., 2004) and longitudinal (Reisenhofer et al., 2019) research suggests that self-efficacy may represent a modifiable intervention target to facilitate change in IPV-related outcomes.

Prior IPV research has examined both coping self-efficacy, which refers to one’s perceived ability to specifically manage the effects of recovering from IPV (DeCou et al., 2015), and general self-efficacy, which refers to a global belief in one’s ability to carry out actions that produce desired effects (Benight & Bandura, 2004). A longitudinal study from the same larger project as the current study (Webermann et al., 2022) found that IPV-related PTSD symptoms among women were associated with more future IPV experiences via reduced general self-efficacy, but self-efficacy did not mediate the effect of IPV experiences on subsequent PTSD symptoms. Other previous studies documenting the role of self-efficacy on the mental health of people who have experienced IPV have been cross-sectional. For example, DeCou et al. (2015) found that IPV coping self-efficacy moderated the association between recent IPV and PTSD symptoms among incarcerated women. Another cross-sectional study of community women experiencing bidirectional IPV found that reduced relationship self-efficacy was associated with greater negative mental health symptoms and the frequency of IPV experiences, but an indirect effect of self-efficacy was only observed for the association between psychological IPV experiences and each of the mental health outcomes (i.e., PTSD, depression, and anxiety; T. P. Sullivan et al., 2013). Together, these studies highlight that self-efficacy may influence psychological symptoms among IPV survivors and that this may vary for IPV subtypes. However, additional research is needed to examine how specific types of IPV experiences may impact self-efficacy and valued living as transdiagnostic aspects of broader psychological health.

The primary objective of the current study was to build upon the predominantly cross-sectional research that has documented the influence of IPV experiences and self-efficacy on mental health symptoms by examining valued living as an important indicator of broader psychological health. We examined this question in a sample of women veterans, with research suggesting that this population is 1.6 times more likely to experience lifetime IPV relative to nonveteran women (Dichter et al., 2014). Moreover, prevalence estimates suggest that 45.7% of women veterans will experience lifetime IPV and 27.9% will experience past-year IPV (Iverson et al., 2024). Thus, women veterans represent a particularly important population for research. In the current study, we conducted a series of path analysis models examining the associations between IPV experiences and valued living, mediated by the effect of self-efficacy, within a longitudinal study of women veterans. We focused on general self-efficacy because trauma exposure can influence self-efficacy across broad life domains (Benight & Bandura, 2004). We separately examined lifetime and recent IPV experiences as well as the unique effects of physical, psychological, and sexual IPV. We hypothesized that a higher frequency of overall IPV experiences would be indirectly linked to less valued living through lower self-efficacy. Given research suggesting that experiencing IPV over longer periods is associated with worse health outcomes (Bonomi et al., 2006), we expected to observe more pronounced effects for lifetime IPV relative to more recent IPV due to the potentially increased detrimental impacts of chronic and frequent IPV experiences on self-efficacy and valued living. We did not specify hypotheses regarding differences for IPV subtypes (i.e., psychological, physical, and sexual) due to a paucity of related research.

## METHOD

### Participants and procedure

Data were drawn from a larger national web-based survey study of 411 women veterans assessed across six time points. The study was initially designed to be a cross-sectional assessment of women’s preferences for IPV care, and five additional assessment points were later added to further assess IPV and health. Participants were recruited from KnowledgePanel, maintained by the Ipsos research firm, which is a probability-based, nonvolunteer access panel composed of 55,000 adults who are representative of 97% of U.S. households. KnowledgePanel participants receive “points” in exchange for participation, which they can redeem for prizes. At the initial time point, all women veterans enrolled in the panel (*N* = 548) were eligible to participate regardless of whether they had experienced IPV. Participants were eligible for subsequent time points if they participated at the previous time point and were still enrolled in the panel. For each survey, Ipsos emailed eligible participants an invitation describing the study purpose, elements of informed consent, and a statement that the individual could participate regardless of whether they experienced an unsafe or unhealthy relationship. All study procedures were approved by the VA Boston Healthcare System Institutional Review Board.

For the current study, we focused on three time points at which the variables of interest were assessed. Thus, the current study sample includes 190 women who participated in the first time point examined (Time 1 [T1]; 72.7% of eligible individuals). The second time point examined (Time 2 [T2]) occurred approximately 3 years following T1 and included 142 women veterans (81.6% of eligible individuals, 74.7% of T1 participants). The third time point examined (Time 3 [T3]) occurred approximately 1 year following T2 and included 101 women veterans (88.6% of eligible individuals, 71.1% of T2 participants, 53.2% of T1 participants). Table 1 displays the sociodemographic characteristics of the study sample. Relative to T1 completers, participants who completed all time points were older, *t*(168.54) = −2.17, *p* = .031, *d* = −0.32, and more represented by non-Hispanic White participants, *χ*^2^(4, *N* = 190) = 9.66, *p* = .047. No differences were observed for household income, educational attainment, sexual identity, marital status, branch of military service, rank at military separation, combat exposure, or military sexual trauma (MST) history, *p*s = .115–.890.

### Measures

#### IPV experiences

The Revised Conflict Tactics Scales (CTS2; Straus et al., 1996) is a self-report measure of IPV that has demonstrated evidence of internal consistency, test–retest reliability, convergent validity, and discriminant validity (Straus et al., 1996; Vega & O’Leary, 2007). We used subscales assessing psychological (eight items), physical (12 items), and sexual (seven items) IPV experiences. During the preceding time point of the larger study, this measure was administered with respect to (a) past-year behavior frequency reports of IPV as well as (b) behavior frequency reports for IPV that occurred before the past year. During the first time point examined in the current study, participants also reported (c) the frequency of behaviors that they experienced in the prior 6 months (i.e., the timeframe since the prior administration of the measure). Participants were asked to indicate how frequently they experienced each act of IPV, rating their responses on a 7-point scale. For each behavior, responses were coded at the midpoint of the range for the endorsed response anchor (i.e., 0 = *never*, 1 = *once*, 2 = *twice*, 4 = *3–5 times*, 8 = *6–10 times*, 15 = *11–20 times*, and 25 = *more than 20 times*). Responses were then summed as a count variable for each assessed timeframe such that higher scores indicated a higher frequency of experiencing the respective type of IPV behaviors. Lifetime IPV as of T1 of the current study reflects a composite of the three aforementioned behavior frequency scores (i.e., denoted previously as (a), (b), and (c)), whereas recent IPV reflects the past–6-month IPV reported at T1 of the current study (i.e., denoted previously as (c)). For both lifetime and recent IPV, composite scores were separately calculated for psychological, physical, sexual, and overall IPV. In the present sample, Cronbach’s alpha values ranged from .85 to .92 for lifetime IPV and from .82 to .91 for recent IPV.

#### Self-efficacy

The General Self-Efficacy Scale (GSS; Schwarzer & Jerusalem, 1995) is a 10-item measure that was used to assess participants’ self-beliefs regarding their ability to cope with an array of life difficulties (e.g., “No matter what comes my way, I’m usually able to handle it”). At T2, participants rated each item on a 4-point Likert response scale ranging from 1 (*not at all true*) to 4 (*exactly true*). Items were summed for a total score (range: 10–40). This measure has demonstrated evidence of construct validity across cultural adaptations (Luszczynska et al., 2005). In the present sample, Cronbach’s alpha was .91.

#### Valued living

The Valued Living Questionnaire (VLQ; Wilson & Groom, 2002; Wilson et al., 2010) is a widely used measure to assess 10 life domains of valued activity: family, intimate relationships, parenting, friendships, work, education, recreation, spirituality, community life, and physical health. At T3, participants rated each domain on a 10-point Likert scale for personal importance (1 = *not at all important* to 10 = *extremely important*) and self-perceptions of past-month consistency of their actions with their value for each domain (1 = *not at all consistent with my value* to 10 = *completely consistent with my value*). For each domain, responses for importance and consistency were multiplied, and the product was then summed across all domains and averaged (range: 10–100), with higher scores indicating higher degrees of valued living. This measure has demonstrated evidence of internal consistency and temporal consistency, as well as adequate validity (Wilson et al., 2010). In the current sample, Cronbach’s alpha was .79.

### Data analysis

We first calculated descriptive statistics and bivariate associations among study variables. We then conducted a series of path analysis models using the *lavaan* (Rosseel, 2012) statistical package in *R* (Version 4.3.0) to examine the effects of IPV at T1 on current valued living at T3, mediated by the effect of self-efficacy at T2. Model 1A examined the effect of lifetime overall IPV reported at T1, and Models 1B–1D examined the effects of lifetime psychological, physical, and sexual IPV at T1, respectively. Model 2A examined the effect of recent (i.e., past–6-months) overall IPV reported at T1, and Models 2B–2D examined the effects of recent psychological, physical, and sexual IPV at T1, respectively. Because these models were just-identified, global fit was perfect, and, thus, fit indices were not interpreted. Consistent with the *N:q rule* (Kline, 2015), we had an appropriate sample size to examine these models with more than 20 participants per parameter. To note, we considered potential theoretically relevant covariates (i.e., combat exposure, MST, age, and race/ethnicity) that may have impacted the observed variables by examining simple linear regressions with each potential covariate as a predictor of either self-efficacy or valued living, but no significant associations were observed. Thus, to favor model parsimony and enhance the replicability of the models in future research, we elected not to include covariates in the proposed models.

Missing data on the GSS at T2 (*n* = 48) and VLQ at T3 (*n* = 89) primarily occurred due to participants being ineligible at that time point (GSS: *n* = 16, VLQ: *n* = 4) or electing not to participate (GSS: *n* = 32, VLQ: *n* = 41). To account for missing data due to attrition across time points, we used full information maximum likelihood given that this method provides more accurate estimates and leverages the entire sample’s observed data without discarding information (see Enders, 2022). Finally, we applied bias-corrected bootstrapping with 5,000 resamples to produce 95% confidence intervals (CIs) to examine the significance of direct and indirect effects. We selected this approach because it is a nonparametric method that accounts for the nonnormal distribution of data and can both maximize power and minimize Type 1 error within smaller samples (Shrout & Bolger, 2002).

## RESULTS

### Descriptive statistics

At T1, 83.6% of participants reported experiencing at least one act of lifetime psychological IPV, and 46.8% of the sample reported at least one act of recent psychological IPV; 52.4% of participants reported experiencing at least one act of lifetime physical IPV, and 11.1% of the sample reported at least one act of recent physical IPV; and 39.7% of participants reported at least one act of lifetime sexual IPV, and 12.1% of the sample reported at least one act of recent sexual IPV. Table 2 displays descriptive statistics and bivariate associations among study variables. As expected, strong positive associations were observed between each of the lifetime IPV variables and between each of the recent IPV variables. Self-efficacy demonstrated small negative associations with lifetime IPV variables and nonsignificant associations with recent IPV variables. Valued living demonstrated small negative associations with all lifetime IPV variables and with recent physical IPV, whereas associations with other recent IPV variables were nonsignificant. Self-efficacy and valued living demonstrated a moderate positive association.

### Mediation models

As presented in Table 3, the results from four separate path analysis models examining lifetime IPV experiences (i.e., overall, psychological, physical, and sexual) suggest that each of the lifetime IPV variables at T1 demonstrated small negative associations with lower self-efficacy at T2 (Path *a*), *β*s = −.20–−.28. Additionally, T2 self-efficacy demonstrated medium positive associations with valued living in each model (Path *b*), *β*s = .39–.41. Across each of the models, the direct effect of the respective IPV variables on valued living was nonsignificant, *β*s −.13–−.19. In each of the models, there were significant indirect effects of the respective lifetime IPV variables at T1 on T3 valued living through T2 self-efficacy, *β*s = −.08–−.11. Overall, the results suggest that lifetime IPV experiences are associated with less valued living through lower self-efficacy.

As presented in Table 4, the results from four separate path analysis models examining recent IPV experiences (i.e., overall, psychological, physical, and sexual) suggest that recent overall, psychological, and sexual IPV variables at T1 demonstrated nonsignificant associations with self-efficacy at T2 (Path *a*), *β*s = −.03–-.17, whereas recent physical IPV demonstrated a large negative association with self-efficacy at T2, (Path *a*), *β* = −.73. Additionally, T2 self-efficacy demonstrated medium positive associations with valued living in each model (Path *b*), *β*s = .36– .43. Across models, the direct effects of recent overall, psychological, and physical IPV variables on valued living were nonsignificant, *β*s = −.14–-.54, whereas recent sexual IPV was directly associated with valued living, *β* = −.27. Indirect effects were nonsignificant in models examining recent overall, psychological, and sexual IPV, whereas the indirect effect of T1 recent physical IPV on T3 valued living through T2 self-efficacy was significant, *β* = −.26. Overall, the results suggest that recent physical IPV may be particularly associated with less valued living through decreased self-efficacy.

To note, we also considered examining the described path models while controlling for immediate prior time points of the mediator and outcome variables. However, these models demonstrated poor global model fit and, thus, were uninterpretable. This may have occurred because we were underpowered to address the added complexity of these models, which would require a sample size of 240 participants to have a ratio of 20 participants per parameter (Kline, 2015). Thus, consistent with our primary aim to examine associations across time points and not infer causal mechanisms, we present our original analyses favoring theory and model parsimony.

## DISCUSSION

The current study builds upon a growing body of predominantly cross-sectional research that has examined self-efficacy as an important factor that may promote or inhibit psychological health among women who have experienced IPV. Our study extends prior work by examining valued living as a transdiagnostic outcome of broader well-being. A strength of the current study is that we leveraged longitudinal data to examine both lifetime and recent IPV experiences along with the unique effects of psychological, physical, and sexual IPV. The findings indicate that lifetime experiences of overall, psychological, physical, and sexual IPV were all indirectly associated with less valued living through lower self-efficacy. However, for recent IPV, this indirect relation was only observed for physical IPV. These findings point to key nuances in the impact that experiencing IPV may have on self-efficacy and valued living. Moreover, the results demonstrate the insidious nature of lifetime IPV exposure and the impact it can have on one’s sense of self and purposeful behavior in important life domains.

The current findings align with prior research suggesting that experiencing IPV may contribute to poorer self-efficacy and psychological health (DeCou et al., 2015; T. P. Sullivan et al., 2013; Webermann et al., 2022) and that valued living may be an important aspect of well-being that is negatively impacted by experiencing interpersonal violence (Cabrera et al., 2021). The chronic and invalidating nature of IPV can be destabilizing for individuals who have experienced IPV and can have widespread effects on a range of psychological processes (e.g., empowerment; Cerulli et al., 2012; Hing et al., 2021), which may compromise one’s ability to engage in life in a meaning-driven or values-aligned way. Living in a less value-aligned way may increase the risk for psychopathology and decrease both resilience to stress and overall quality of life (Ceary et al., 2019; Tunç et al., 2023). Additionally, the findings suggest that behavioral activation in personally valued life domains may be an important way to enhance well-being in the wake of traumatic experiences (Jakupcak et al., 2010). Consistent with theories underlying some psychosocial interventions for IPV, including advocacy, individuals may feel a lack of control and empowerment due to experiencing IPV-related stressors (e.g., ongoing safety threats, financial concerns, child care) and health consequences (e.g., injuries, emotional distress); increasing a sense of efficacy and purposefulness in areas of life that are within their control may improve well-being (Doyle et al., 2022; C. M. Sullivan, 2018).

Importantly, lifetime IPV experiences consistently demonstrated negative associations with valued living through less self-efficacy regardless of which type of IPV participants experienced (i.e., psychological, physical, or sexual). This is consistent with prior research demonstrating poor psychological health outcomes across all IPV subtypes (Dichter et al., 2014). Therefore, the current findings may further highlight that the chronicity of IPV exposure over time has consequences for psychological health and well-being, particularly with respect to self-efficacy and valued living.

In contrast, we observed that the effects of recent overall IPV experiences did not have a similar indirect effect on current valued living through self-efficacy. This held true for both recent psychological and recent sexual IPV. However, there was an indirect effect that emerged for recent experiences of physical IPV. In conjunction with the findings for lifetime IPV experiences, this may suggest that more acute experiences of psychological and sexual IPV play a less prominent role in self-efficacy and valued living over time, further highlighting that the chronicity of exposure to these forms of IPV may be particularly important for well-being. In contrast, recent physical IPV was associated with less valued living through decreased self-efficacy. This may align with prior work suggesting that stress, challenges, and coping efforts related to managing ongoing safety threats may interfere with daily functioning and be deleterious to well-being (Cerulli et al., 2012). This finding further supports that establishing physical safety is important to the protection of short- and long-term health and well-being of individuals who experience IPV (Kahraman & Bell, 2017).

These results have implications for prevention and intervention efforts. Prior literature suggests that self-efficacy is a protective factor that can potentially buffer against the detrimental effects of IPV (Crapolicchio et al., 2021; T. P. Sullivan et al., 2013) and is a key mechanism for promoting recovery (C. M. Sullivan, 2018). Yet, a recent meta-analytic review suggests that many existing psychosocial interventions are often ineffective at increasing self-efficacy among IPV survivors (Karakurt et al., 2022). A recently developed intervention, Recovering from IPV through Strengths and Empowerment (RISE; Iverson et al., 2021), is a transdiagnostic and flexible treatment for individuals with current or recent IPV experiences, with an emphasis on helping patients identify personalized goals that align with their values and making actionable changes. Importantly, evidence suggests that this intervention can effectively increase both self-efficacy and valued living (Iverson et al., 2022), suggesting that this may be a promising early intervention to prevent the long-term negative impact that experiencing IPV can have on well-being. Other psychosocial interventions that may address self-efficacy and valued living in the context of specific mental health needs (e.g., PTSD) among individuals who have experienced IPV have also been developed, such as Helping Overcome PTSD through Empowerment (HOPE; Johnson et al., 2011, 2016). Despite the potential benefits of targeting self-efficacy and valued living as important transdiagnostic psychological processes following IPV, gaps remain with respect to the accessibility of evidence-based psychosocial interventions within real-world clinical settings, and further work is needed to advance implementation and dissemination efforts.

The current study had limitations. First, this study reflects the experiences of a community sample of women veterans, which may limit the generalizability of the findings to other populations of individuals who experience IPV. Additionally, the larger study from which these data were drawn was not initially designed to examine the current research question. Thus, we were unable to examine additional variables that may inform the observed results, such as additional relationship characteristics, IPV across multiple relationships, and contextual factors. Finally, due to the relatively small sample size, we were likely underpowered to examine additional variables in the model that would have allowed us to examine the effects of IPV above and beyond other predictors, as well as autoregressive controls for self-efficacy and valued living, which may have introduced some bias in the observed effects. Thus, we are unable to infer causality and determine whether the directions of the associations among IPV, self-efficacy, and valued living occur reciprocally over time. Indeed, many women who experience IPV have experienced multiple forms of interpersonal violence across the lifespan (Dugal et al., 2018; Iverson et al., 2013), and decreased self-efficacy and valued living may both be exacerbated by past experiences of violence and contribute to the risk of experiencing future instances of violence.

Future research may address these limitations by examining these constructs among larger and more diverse samples of individuals who have experienced IPV, including nonveterans, men, and/or individuals with minoritized gender and sexual identities. Additionally, individuals with minoritized identities (e.g., sexual and gender minority individuals, Black women) may experience IPV at higher rates (Iverson et al., 2024) and may experience additional forms of disempowerment and chronic invalidation due to marginalized social identities that further compromise self-efficacy (C. M. Sullivan, 2018). Thus, an examination of intersecting experiences is critically needed to advance future IPV research. Similarly, future research may examine how the cumulative effects of trauma and specific mental health conditions (e.g., PTSD, depression) impact the observed results. Additionally, further longitudinal research specifically designed to infer causal relations, observe whether the variables are reciprocally related over time, and examine within- and between-person change is needed. Larger sample sizes are particularly needed to capture more variability in the extent and types of IPV experienced and sociodemographic characteristics to further elucidate how these constructs are influenced by IPV over time.

In conclusion, valued living is an important transdiagnostic component of well-being that may be compromised due to the detrimental effects of IPV on self-efficacy. Although the findings need replication, they highlight that all forms of lifetime IPV may impact these outcomes, whereas only recent physical IPV was associated with self-efficacy and valued living. Psychosocial interventions that directly address self-efficacy and valued living may help optimize, as early as possible, psychological health and well-being after experiencing IPV.

## Figures and Tables

**TABLE 1:** Sociodemographic and military characteristics of the study sample

Characteristic	*M*	*SD*
Age (years)	54.06	14.00
	*n*	%

Race/ethnicity		
Non-Hispanic White	137	72.1
Non-Hispanic Black	22	11.6
Hispanic	21	11.1
Other racial or ethnic identities	10	5.3

Sexual identity		
Heterosexual	170	90.4
Lesbian, gay, bisexual, or uncertain	18	9.6

Education		
Bachelor’s degree or higher	86	45.3
Some college or an associate’s degree	82	43.1
High school or less	22	11.6

Marital status		
Married	105	55.3
Living with a partner	13	6.8
Divorced	41	22.0
Separated	5	2.6
Widowed	8	4.2
Never married	18	10

Branch of service		
Army	77	41.2
Air Force	49	26.2
Navy	47	25.1
Marines	9	4.8
Coast Guard	5	2.7

Endorsed military sexual trauma	87	45.8

Endorsed combat exposure	22	11.6

**TABLE 2:** Descriptive statistics and bivariate associations among study variables

Variable	1	2	3	4	5	6	7	8	9	10
1. T1 lifetime overall IPV	–	.54***	.90***	.55***	.92***	.47***	.88***	.39***	−.24**	−.30**
2. T1 recent overall IPV		–	.40***	.85***	.55***	.92***	.57***	.91***	−.05	−.20*
3. T1 lifetime psychological IPV			–	.57***	.70***	.25***	.64***	.18*	−.20*	−.25*
4. T1 recent psychological IPV				–	.42***	.61***	.44***	.61***	−.02	−.13
5. T1 lifetime physical IPV					–	.59***	.88***	.47***	−.21*	−.29**
6. T1 recent physical IPV						–	.56***	.87***	−.16	−.24*
7. T1 lifetime sexual IPV							–	.54***	−.25**	−.29*
8. T1 recent sexual IPV								–	−.04	−.08
9. T2 self-efficacy									–	.43***
10. T3 valued living										–
Cronbach’s *α*	.85	.90	.87	.82	.86	.91	.92	.90	.91	.79
*M*	66.24	10.61	41.76	6.66	14.00	2.09	10.49	1.85	31.81	52.40
*SD*	132.85	35.74	65.42	15.36	47.16	13.95	34.13	10.83	4.62	21.61
Observed range	0–1,083	0–194	0–371	0–65	0–418	0–109	0–294	0–46	18–40	1.6–94.2

*Note*: IPV = intimate partner violence; T1 = Time 1; T2 = Time 2; T3 = Time 3.

* *p* < .05.

** *p* <.01.

*** *p* <.001.

**TABLE 3:** Path analysis models examining associations between lifetime intimate partner violence (IPV) experiences and valued living through self-efficacy

Model	Self-efficacy (*R*^2a^)	Valued living (*R*^2a^)	*B*	95% CI	*β*	95% CI
Model 1A: Overall IPV	.07	.23				
Outcome: T2 self-efficacy						
T1 IPV			**−** **0.01**	**[**−**0.02, −0.004]**	**−** **.26**	**[**−**.42,** −**.10]**
Outcome: T3 valued living						
T1 IPV			−0.03	[−0.05, 0.01]	−.18	[−.33, .05]
T2 self-efficacy			**1.83**	**[0.72, 2.74]**	**.39**	**[.17, .58]**
Indirect effect			**−** **0.02**	**[**−**0.04,** −**0.01]**	**−** **.10**	**[**−**.19,** −**.03]**
Total effect			**−** **0.05**	**[**−**0.07,** −**0.01]**	**−** **.28**	**[**−**.42,** −**.06]**
Model 1B: Psychological IPV	.04	.21				
Outcome: T2 self-efficacy						
T1 IPV			**−** **0.02**	**[**−**0.03,** −**0.001]**	**−** **.20**	**[**−**.37,** −**.02]**
Outcome: T3 valued living						
T1 IPV			−0.04	[−0.09, 0.03]	−.13	[−.30, .08]
T2 self-efficacy			**1.91**	**[0.85, 2.82]**	**.41**	**[.19, .59]**
Indirect effect			**−** **0.03**	**[**−**0.06,** −**0.004]**	**−** **.08**	**[**−**.17,** −**.01]**
Total effect			**−** **0.07**	**[**−**0.12,** −**0.002]**	**−** **.21**	**[**−**.38,** −**.01]**
Model 1C: Physical IPV	.06	.24				
Outcome: T2 self-efficacy						
T1 IPV			**−** **0.02**	**[**−**0.05,** −**0.01]**	**−** **.24**	**[**−**.42, −.11]**
Outcome: T3 valued living						
T1 IPV			−0.09	[−0.21, 0.02]	−.19	[−.37, .02]
T2 Self-Efficacy			**1.87**	**[0.82, 2.76]**	**.40**	**[.18, .59]**
Indirect effect			**−** **0.04**	**[**−**0.10,** −**0.01]**	**−** **.10**	**[**−**.19,** −**.03]**
Total effect			**−** **0.13**	**[**−**0.25,** −**0.05]**	**−** **.29**	**[**−**.45,** −**.10]**
Model 1D: Sexual IPV	.08	.24				
Outcome: T2 self-efficacy						
T1 IPV			**−** **0.04**	**[**−**0.09,** −**0.03]**	**−** **.28**	**[**−**.53,** −**.17]**
Outcome: T3 valued living						
T1 IPV			−0.12	[−0.26, 0.09]	−.19	[−.36, .08]
T2 self-efficacy			**1.84**	**[0.77, 2.75]**	**.39**	**[.17, .58]**
Indirect effect			**−** **0.07**	**[**−**0.17,** −**0.02]**	**−** **.11**	**[**−**.24,** −**.05]**
Total effect			**−** **0.20**	**[**−**0.38,** −**0.07]**	**−** **.31**	**[**−**.49,** −**.07]**

*Note*: Bolding indicates a significant result. T1 = Time 1; T2 = Time 2; T3 = Time 3; CI = confidence interval.

a Reflects explained variance in respective variables.

**TABLE 4:** Path analysis models examining associations between recent intimate partner violence (IPV) experiences and valued living through self-efficacy

Model	Self-efficacy (*R*^2a^)	Valued living (*R*^2a^)	*B*	95% CI	*β*	95% CI
Model 1A: Overall IPV	.03	.38				
Outcome: T2 self-efficacy						
T1 IPV			−0.02	[−0.11, 0.04]	−.17	[−.71, .28]
Outcome: T3 valued living						
T1 IPV			−0.29	[−0.67, 0.17]	−.42	[−.77, .26]
T2 self-efficacy			**1.98**	**[1.00, 2.83]**	**.38**	**[.18, .59]**
Indirect effect			−0.04	[−0.25, 0.08]	−.06	[−.33, .11]
Total effect			−0.33	[−0.75, 0.15]	−.48	[−.84, .18]
Model 1B: Psychological IPV	.001	.21				
Outcome: T2 self-efficacy						
T1 IPV			−0.01	[−0.12, 0.06]	−.03	[−.40, .19]
Outcome: T3 valued living						
T1 IPV			−0.19	[−0.63, 0.36]	−.14	[−.44, .24]
T2 self-efficacy			**2.05**	**[1.03, 2.91]**	**.43**	**[.23, .61]**
Indirect effect			−0.02	[−0.27, 0.13]	−.01	[−.19, .09]
Total effect			−0.21	[−0.65, 0.38]	−.15	[−.46, .21]
Model 1C: Physical IPV	.53	.71				
Outcome: T2 self-efficacy						
T1 IPV			**−** **0.35**	**[**−**0.57,** −**0.09]**	**−** **.73**	**[**−**0.90,** −**.13]**
Outcome: T3 valued living						
T1 IPV			−1.37	[−2.79, 2.03]	−.54	[−.75, .78]
T2 self-efficacy			**1.94**	**[0.98, 2.81]**	**.36**	**[.17, .63]**
Indirect effect			**−** **0.67**	**[**−**1.45,** −**0.21]**	**−** **.26**	**[**−**.47,** −**.04]**
Total effect			−2.04	[−3.53, 1.77]	−.80	[−.93, .60]
Model 1D: Sexual IPV	.03	.29				
Outcome: T2 self-efficacy						
T1 IPV			−0.07	[−0.92, 0.003]	−.16	[−.60, .05]
Outcome: T3 valued living						
T1 IPV			**−** **0.56**	**[**−**8.93,** −**0.29]**	**−** **.27**	**[**−**.73,** −**.04]**
T2 self-efficacy			**2.09**	**[1.12, 2.97]**	**.43**	**[.20, .60]**
Indirect effect			−0.15	[−2.09, .003]	−.07	[−.23, .02]
Total effect			**−** **0.71**	**[**−**10.28,** −**0.34]**	**−** **.34**	**[**−**.87,** −**.05]**

*Note*: Bolding indicates a significant result. T1 = Time 1; T2 = Time 2; T3 = Time 3; CI = confidence interval.

a Reflects explained variance in respective variables.
